# The Small RNA NcS25 Regulates Biological Amine-Transporting Outer Membrane Porin BCAL3473 in Burkholderia cenocepacia

**DOI:** 10.1128/msphere.00083-23

**Published:** 2023-03-27

**Authors:** Andrea M. Sass, Tom Coenye

**Affiliations:** a Laboratory of Pharmaceutical Microbiology, Ghent University, Ghent, Belgium; University of Nebraska Medical Center

**Keywords:** *Burkholderia*, small RNA, biofilms, porins

## Abstract

Regulation of porin expression in bacteria is complex and often involves small-RNA regulators. Several small-RNA regulators have been described for Burkholderia cenocepacia, and this study aimed to characterize the biological role of the conserved small RNA NcS25 and its cognate target, outer membrane protein BCAL3473. The B. cenocepacia genome carries a large number of genes encoding porins with yet-uncharacterized functions. Expression of the porin BCAL3473 is strongly repressed by NcS25 and activated by other factors, such as a LysR-type regulator and nitrogen-depleted growth conditions. The porin is involved in transport of arginine, tyrosine, tyramine, and putrescine across the outer membrane. Porin BCAL3473, with NcS25 as a major regulator, plays an important role in the nitrogen metabolism of B. cenocepacia.

**IMPORTANCE** Burkholderia cenocepacia is a Gram-negative bacterium which causes infections in immunocompromised individuals and in people with cystic fibrosis. A low outer membrane permeability is one of the factors giving it a high level of innate resistance to antibiotics. Porins provide selective permeability for nutrients, and antibiotics can also traverse the outer membrane by this means. Knowing the properties and specificities of porin channels is therefore important for understanding resistance mechanisms and for developing new antibiotics and could help in overcoming permeability issues in antibiotic treatment.

## INTRODUCTION

The outer membrane (OM) is a defining characteristic of Gram-negative bacteria. It provides a permeability barrier for hydrophilic and amphiphilic molecules and thereby acts as a point of control between bacterial cells and their environment. Nutrients and other essential molecules can traverse the OM through the lipid bilayer or through water-filled protein channels, the porins. There are two major functional classes of porins, nonspecific general porins and substrate-specific channels. Enterobacteria have a large number of nonspecific general porins and very few specific channels (1, 2), whereas Pseudomonas aeruginosa and Acinetobacter baumannii have a large set of substrate-specific channels and general porins with low pore-forming activity (“slow porins”) (3, 4). The absence of large-channel general porins has been implicated as one of the causes for the high innate resistance to antibiotics of these two bacterial species (3–7).

Burkholderia cenocepacia, a betaproteobacterium, is an opportunistic pathogen known for its large genome and versatile metabolism and for its ability to colonize a large range of environmental niches, from the rhizosphere to the lungs of immunocompromised individuals (8, 9). It is innately resistant to most antibiotics (10). B. cenocepacia genomes encode a large family of homologous porins with, depending on genome size, 35 to 50 members (11, 12) with unknown specificity. Homologs of general diffusion porins, such as Escherichia coli OmpF and OmpC, the P. aeruginosa slow porin OprF (13), and the A. baumannii slow porin OmpA (7), are absent from the B. cenocepacia genome. The specific porins of the OprD family, which have 19 members in P. aeruginosa PAO1, are represented by only one homolog in B. cenocepacia. The large family of porin proteins might therefore be important for specific nutrient uptake in B. cenocepacia.

The regulation of porin expression is complex (1, 4), involving an interplay of DNA binding regulators, sigma factors, and small noncoding regulatory RNAs (sRNAs). Bacterial sRNAs play diverse physiological roles in the response to stress and the regulation of metabolism, fine-tuning gene expression usually at the posttranscriptional level (14, 15). The canonical mode of action of sRNAs is interaction with an mRNA complement, often at the Shine-Dalgarno (SD) sequence. The sRNA-mRNA complex is usually stabilized by RNA binding proteins such as Hfq. sRNA-mRNA interaction at the SD sequence interferes with translation initiation, and untranslated mRNA is quickly degraded (14, 16).

Enterobacteria use many sRNAs to control the porin composition of the OM, with some overlap and redundancy between the porin targets of different sRNAs (1, 17–19). For P. aeruginosa, only one porin-regulating sRNA has been described to date (20, 21). B. cenocepacia contains numerous sRNAs and two Hfq protein homologs (11, 22). In B. cenocepacia J2315, sRNAs have a role in regulating the response to iron depletion (23) and in regulating carbon substrate utilization (24).

Here, we present the characterization of sRNA NcS25, highly abundant in biofilm-grown B. cenocepacia (22), and its target, porin protein BCAL3473.

## RESULTS

### Outer membrane porins are conserved predicted targets of NcS25.

**FIG 1:**
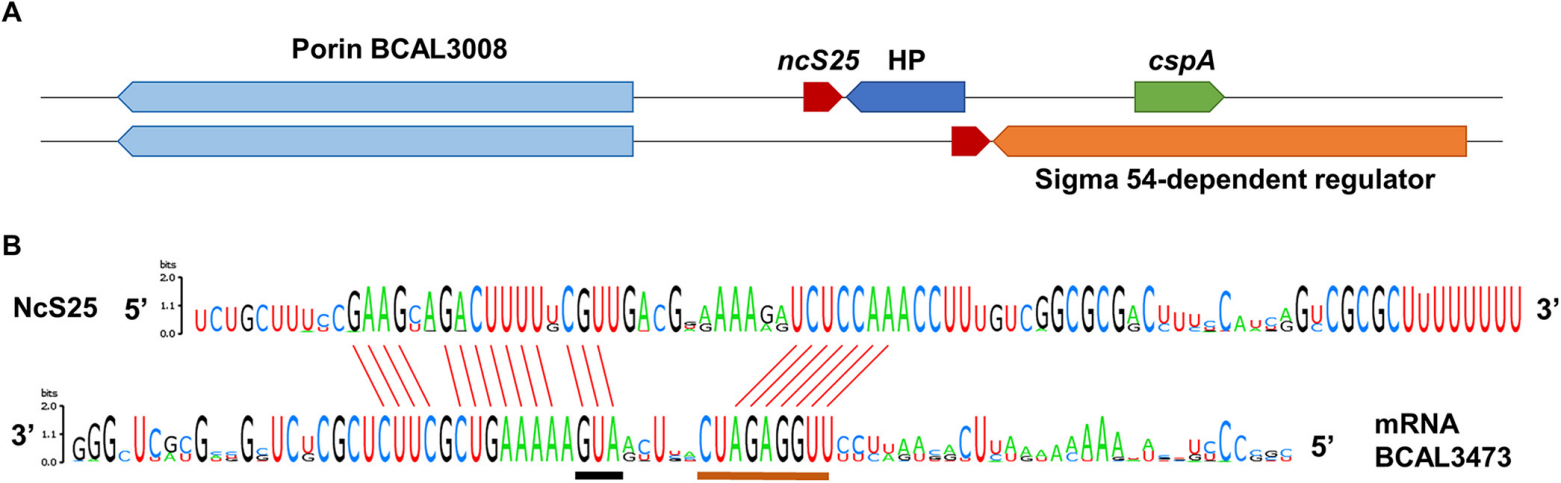
Conservation of genomic context and interaction region of *ncS25* in *Burkholderia* spp., *Paraburkholderia* spp., and *Pandoraea* spp. (A) A porin homologous to BCAL3008 is encoded upstream of *ncS25* in all genomes investigated. Encoded downstream are a hypothetical protein (HP) and an RNA-binding cold shock protein (CspA) or a σ^54^-dependent regulator. *ncS25* shares its terminator sequence with the downstream gene. (B) Consensus sequence and conservation of the interaction region. The full NcS25 sequence (top) and part of the 5′ UTR and coding sequence of porin gene BCAL3473 (bottom) are shown. Conserved predicted paired bases are indicated with red bars between the sequence logos. The start codon is underlined in black and the Shine-Dalgarno sequence in brown.

NcS25 is an 84-nucleotide (nt) noncoding RNA, as determined by differential transcriptome sequencing (dRNA-Seq) (22), and *ncS25* is located in the intergenic region between a gene encoding a conserved hypothetical protein (BCAL3007) and a gene encoding a porin protein (BCAL3008) (Fig. 1A) (22). *ncS25* and its upstream genomic context are conserved within the genera *Burkholderia*, *Paraburkholderia*, and *Pandoraea*. Located downstream of the *ncS25* sequence is a gene annotated as hypothetical protein with which it shares a bidirectional rho-independent terminator, followed by a gene annotated as RNA-binding chaperone protein, or in *Pandoraea* spp. a gene annotated as a sigma 54 (σ^54^)-dependent Fis family transcriptional regulator (Fig. 1A).

Ten genomes each of the genera *Burkholderia*, *Paraburkholderia*, and *Pandoraea* were computationally screened for conserved putative targets of NcS25 using CopraRNA (25). This software tool takes the energy required to unfold the secondary structure of sRNA and target as well as conservation of targets into account and combines the results with phylogenetic information (25). The screening was limited to the region 200 nt upstream to 100 nt downstream of gene translation start sites, and the output comprised hits conserved among at least 50% of input strains. In the genus *Burkholderia*, the 100 hits with the highest probability included 14 genes encoding porin proteins, 13 of which belonged to the large family of homologous porin proteins found in *Burkholderia* spp. The gene with the most extensive interaction region was porin BCAL3473, a member of this porin protein family.

The combined CopraRNA *P* value for the predicted interaction between NcS25 and the BCAL3473 homolog in *Burkholderia* spp. is 4 orders of magnitude lower than for the next-likeliest interaction predicted by this tool (see Table S1 in the supplemental material), setting this particular predicted target apart from the rest. This is similar for the respective BCAL3473 homologs in *Paraburkholderia* spp. and *Pandoraea* spp. (Table S1).

10.1128/msphere.00083-23.1 TABLE S1 The 20 putative targets with the lowest combined *P* value as computed by nCopraRNA, by genus (separate Excel file). Download Table S1, XLSX file, 0.03 MB. Copyright © 2023 Sass and Coenye. 2023 Sass and Coenye. https://creativecommons.org/licenses/by/4.0/ This content is distributed under the terms of the Creative Commons Attribution 4.0 International license.

The putative SD sequence of BCAL3473 is conserved among all genera which harbor *ncS25*, and in all cases, a 7-nt stretch within the SD sequence has a perfect complement within its cognate NcS25 (Fig. 1B; Fig. S1). Another interaction region is predicted for the start of the BCAL3473 coding sequence; here, the complementarity is not always perfect. The region between the SD sequence and the translation start site of BCAL3473 is not conserved, nor is the rest of the 5′ untranslated region (UTR).

10.1128/msphere.00083-23.5 FIG S1 Sequence conservation of NcS25 among *Pandoraea* spp., *Burkholderia* spp., and *Paraburkholderia* spp. The interaction site complementary to the SD region is underlined. The height of the grey bars under the alignment denotes sequence conservation in the respective column. The alignment was computed with LocARNA (50). Download FIG S1, TIF file, 2.5 MB. Copyright © 2023 Sass and Coenye. 2023 Sass and Coenye. https://creativecommons.org/licenses/by/4.0/ This content is distributed under the terms of the Creative Commons Attribution 4.0 International license.

**FIG 2:**
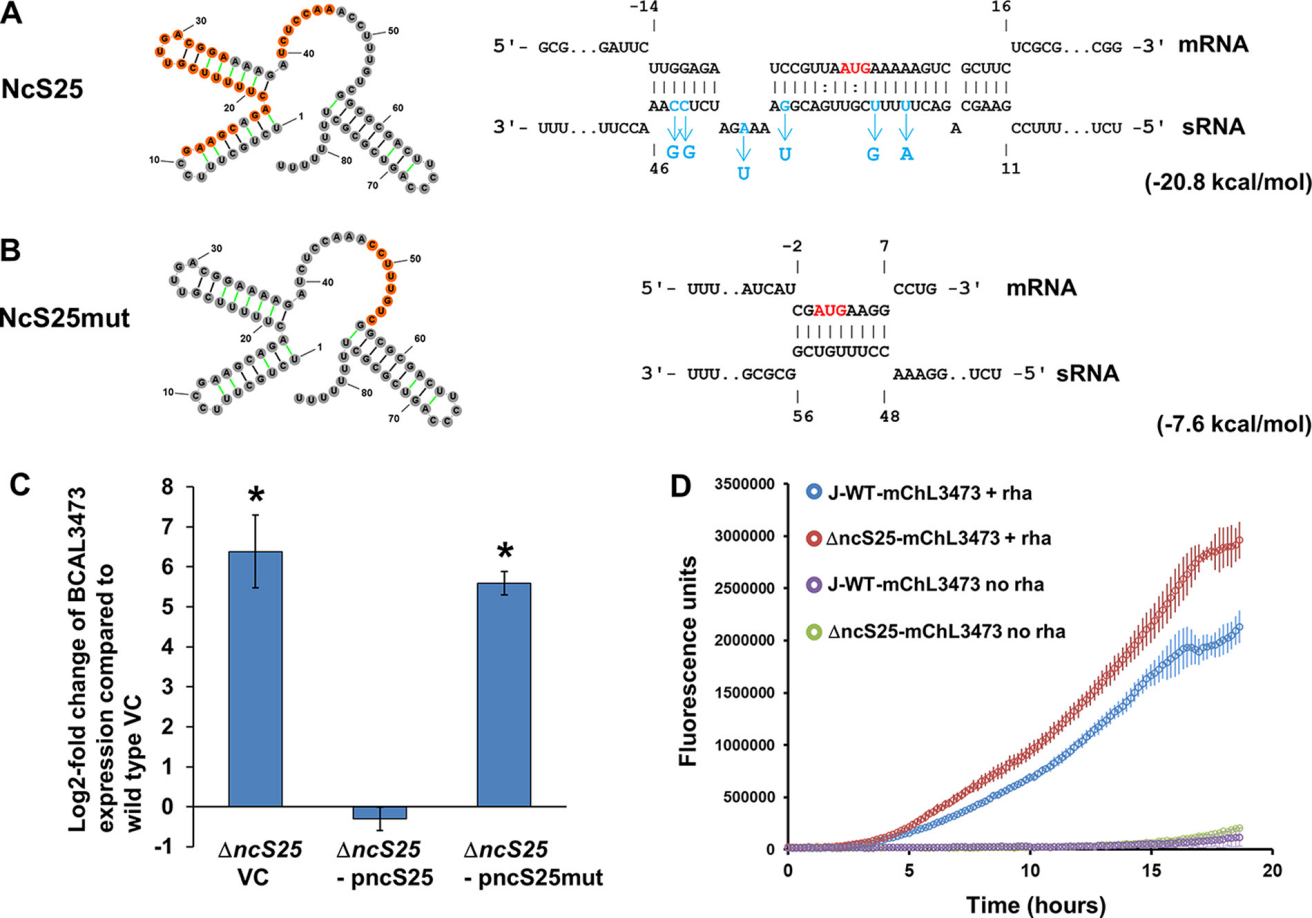
Repression of porin BCAL3473 by NcS25 depends on the integrity of the predicted interaction region. (A and B) Secondary structure and base-pairing with BCAL3473 mRNA of NcS25 and NcS25mut, a derivative of NcS25 with point mutations in the predicted interaction region. The predicted secondary structure is identical for both sequences, whereas the predicted interaction with the BCAL3473 mRNA (indicated in orange on the secondary structure) is much shorter for NcS25mut than for native NcS25. The part of NcS25 interacting with the SD sequence is situated in a single-stranded section and can serve as a seed region for interaction initiation. The translation start site is depicted in red. Nucleotides exchanged in *ncS25* to create *ncS25*mut are indicated in blue. Mutations were chosen to have no impact on the computationally predicted secondary structure of the sRNA. Numbers indicate the position relative to the translation start site for mRNA and sRNA nucleotide position. The predicted free energy of the interaction is shown in parentheses. (C) Expression of BCAL3473 in the Δ*ncS25* mutant, compared to wild-type J2315, is strongly increased in planktonic cultures grown in rich mineral medium. This was complemented by expression of the native NcS25 in *trans*, whereas in *trans* expression of NcS25mut did not have this effect. Error bars depict standard deviations, and asterisks denote statistically significant changes (*P* ≤ 0.05) compared to wild-type B. cenocepacia J2315 vector control (VC). The data are derived from three biological replicates with two technical replicates each. (D) Fluorescence produced by translational reporter gene fusion confirms regulation of BCAL3473 by NcS25. The 5′ UTR and first 84 nt of the coding sequence of BCAL3473 were fused to mCherry and the construct transformed into wild-type B. cenocepacia and the Δ*ncS25* mutant. Incubation was performed with and without 0.1% rhamnose (rha). The error bars represent the standard deviations for three biological replicates.

BCAL3473 is 383 amino acids long with a size of 39.91 kDa, and it forms a 16-stranded beta-barrel to span the OM. The 5′ UTR of BCAL3473 is 87 bases long (26). The predicted interaction region in B. cenocepacia spans from nucleotide position −14 in the 5′ UTR of BCAL3473 to 16 nt into the beginning of the gene (Fig. 2A).

### sRNA NcS25 represses expression of porin BCAL3473.

To confirm the regulation of targets by NcS25, an *ncS25* deletion mutant of B. cenocepacia J2315 (Δ*ncS25*) was constructed. The Δ*ncS25* strain had no growth defect compared to the wild type in rich mineral medium (Fig. S2).

10.1128/msphere.00083-23.6 FIG S2 Growth of B. cenocepacia J2315 wild type and mutants on arginine and putrescine as sole sources of nitrogen. Deletion of porin BCAL3473 attenuates growth on both compounds, which could be complemented by expressing BCAL3473 in *trans*. Deletion of *ncS25* accelerated growth on putrescine, partially complemented by expressing NcS25 in *trans*. Growth on single compounds as nitrogen sources was tested in a mineral medium without ammonium and with 25 mM glucose as carbon source. Arginine and putrescine were added at a concentration of 2 mM. To test for growth defects, a rich mineral medium was used (right side panels). The growth curves shown are representative of a minimum of three biological replicates. WT, wild type; VC, vector control. Download FIG S2, TIF file, 1.1 MB. Copyright © 2023 Sass and Coenye. 2023 Sass and Coenye. https://creativecommons.org/licenses/by/4.0/ This content is distributed under the terms of the Creative Commons Attribution 4.0 International license.

The expression of six putative targets was analyzed by qPCR, comparing the wild type and deletion mutant in planktonic culture and in biofilms. Besides porin BCAL3473, three other porins with a similar predicted sRNA-mRNA interaction that included the region around the gene start site were tested (BCAL2615, BCAM1787, and BCAL0594), as well as the putative target porin encoded adjacent to *ncS25* (BCAL3008) and a phosphotransferase system II (PTSII) transport protein (BCAL0781) (Fig. S3).

10.1128/msphere.00083-23.7 FIG S3 Interactions for NcS25 with selected predicted target mRNAs. The NcS25 sequence is depicted in blue. Numbers in parentheses denote the mRNA nucleotide position of interactions, relative to the translation start site (in red); the predicted change in free energy of an interaction is given below. Download FIG S3, TIF file, 0.8 MB. Copyright © 2023 Sass and Coenye. 2023 Sass and Coenye. https://creativecommons.org/licenses/by/4.0/ This content is distributed under the terms of the Creative Commons Attribution 4.0 International license.

Of these genes, only BCAL3473 was significantly upregulated in the Δ*ncS25* mutant, and quite considerably, 83-fold in planktonic cultures and 27-fold in biofilms (Table 1). This could be complemented by expressing NcS25 in *trans* from its native promoter (Fig. 2C). When a plasmid with a mutated derivative of *ncS25* in which several bases in the putative interaction region were exchanged was expressed in *trans* (Fig. 2A and B), complementation did not occur (Fig. 2C). Expression of NcS25 in *trans* in the Δ*ncS25* mutant was confirmed by qPCR and was comparable to wild-type expression, for plasmid pncS25 as well as for pncS25mut (quantification cycle [*C_q_*] values: 20.1 for wild type, 19.1 for the Δ*ncS25* mutant carrying pncS25, and 18.5 for the Δ*ncS25* mutant carrying pncS25mut).

**TABLE 1:** Log_2_ fold changes in expression compared to the control condition^*a*^

Gene function^*b*^	Locus tag	Log_2_ fold change in expression^*c*^				
		Δ*ncS25* mutant		N depletion in:		BCAL0461 knockdown^*d*^
		Planktonic	Biofilm	Wild-type J2315	J2315 Δ*ncS25*	
Predicted targets						
Porin	BCAL3473	6.4 (0.91)	4.8 (1.26)	3.3 (0.49)	1.2 (0.19)	−4.7 (0.33)
Porin	BCAL3008	NS	NS	NS	NS	−1.3 (0.11)
Porin	BCAL2615	NS	NS	2.2 (0.04)	1.2 (0.13)	−2.0 (0.49)
Porin	BCAM1787	NS	NS	NS	NS	−2.0 (0.21)
Porin	BCAL0594	NS	NS	NS	NS	−1.0 (0.45)
*nagE*	BCAL0781	NS	NS	NS	NS	NS
sRNA	NcS25	ND	ND	NS	ND	NS
LysR-type regulator	BCAL0461	—	NS	NS	NS	−4.5 (0.54)
Urea transporter	BCAL3098	—	—	9.7 (0.46)	7.0 (0.10)	NS
*ntrB*	BCAL0729	—	—	13.9 (0.42)	11.6 (0.19)	NS

a The control condition was nitrogen-replete medium.

b *nagE* encodes an *N*-acetyl-d-glucosamine phosphotransferase system transporter; *ntrB* encodes nitrogen regulatory protein P-II.

c ND, not detected (*C_q_* value equal to background signal of no reverse transcriptase control); NS, not significant; —, not determined. Values in parentheses are standard deviations for three biological replicates with two technical replicates each.

d In rich medium, compared to the wild type.

Experiments with translational reporter gene fusions to confirm regulation of gene expression with sRNA are usually performed with a plasmid system in which both sRNA and target are overexpressed from separate promoters. In B. cenocepacia J2315, the high innate antibiotic resistance renders most resistance markers unsuitable; moreover, only one useable promoter system and compatible plasmid origin of replication are available. For that reason, only the putative target region was overexpressed, fused to mCherry, and under the control of a rhamnose-inducible promoter. This construct was then transformed into wild-type J2315 and J2315 Δ*ncS25*.

Fluorescence production was slightly accelerated in the Δ*ncS25* strain compared to the wild type (Fig. 2D), confirming the regulation of BCAL3473 by NcS25. In positive controls with the intact mCherry gene, fluorescence production was equal in both the wild-type and Δ*ncS25* strains (Fig. S4).

10.1128/msphere.00083-23.8 FIG S4 Controls for translational reporter gene assay. (Left) Fluorescence produced from the entire intact mCherry gene, with and without 0.1% rhamnose (rha) in the medium (positive control). (Right) Fluorescence produced from the construct used for fusions, without the BCAL3473 gene fragment (negative control). WT, wild type. The error bars represent the standard deviations for three biological replicates. Download FIG S4, TIF file, 0.9 MB. Copyright © 2023 Sass and Coenye. 2023 Sass and Coenye. https://creativecommons.org/licenses/by/4.0/ This content is distributed under the terms of the Creative Commons Attribution 4.0 International license.

### Nitrogen depletion induces expression of porin BCAL3473.

Screening the literature for expression data for porin BCAL3473 revealed that the porin was downregulated when σ^54^ factor enhancer binding protein NtrC was deleted in B. cenocepacia H111 (27) and upregulated in Burkholderia glumae when the regulator GvmR (homolog to BCAL0461) was deleted (28). To explore whether these factors also play a role in BCAL3473 regulation in B. cenocepacia J2315, we investigated expression of NcS25, BCAL3473, and other putative targets under conditions of nitrogen depletion and in a BCAL0461 knockdown mutant.

Under nitrogen depletion, of the tested genes, only those encoding porins BCAL3473 and BCAL2615 were upregulated (Table 1), in the wild-type and Δ*ncS25* strains. As a positive control for response to nitrogen depletion, expression of two genes known to be induced by nitrogen depletion and exhibiting a σ^54^ binding site (29), a urea ABC transporter (BCAL3098), and nitrogen regulatory protein P-II (BCAL0729), was also assessed. As expected, these genes were strongly upregulated under nitrogen depletion (Table 1), whereas NcS25 did not change expression under this condition.

**FIG 3:**
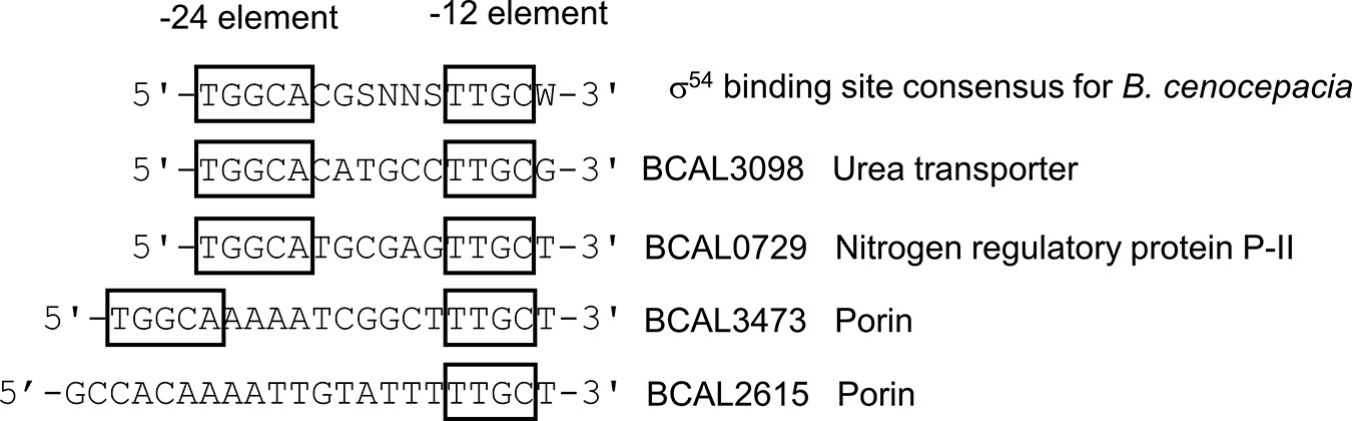
Two-block promoter elements of the σ^54^ binding site of B. cenocepacia. The promoter region of BCAL3473 displays similarities to the σ^54^ binding site consensus. Sequences shown are located up to 14 nucleotides upstream of the transcription start site. Boxes indicate conserved core recognition sequences of the σ^54^ promoter.

The promoter region of porin BCAL3473 contains sequences identical to the −12 and −24 elements of the wider consensus sequence for the σ^54^ binding sites of B. cenocepacia (29), but with an extended spacer sequence (Fig. 3). The promoter region of BCAL2615 contains only the tetranucleotide of the −12 element. No sequences similar to these motifs were detected upstream of *ncS25* or the other quantitative PCR (qPCR)-tested targets.

Knocking down expression of LysR-type regulator BCAL0461 affected the expression of all tested porins, with porin BCAL3473 downregulated to a large extent (Table 1). NcS25 was not differentially expressed in the knockdown mutant (Table 1).

### Biological amines are among the putative substrates of porin BCAL3473.

Porin BCAL3473 was deleted from the genomes of B. cenocepacia strains J2315 and K56-2, and complementation mutants were constructed for expressing BCAL3473 in *trans* under the control of a rhamnose-inducible promoter (Table 2). In rich mineral medium, mutants had no growth defect compared to the respective wild-type strain (Fig. S2).

**TABLE 2:** Strains and plasmids used in this study

Strain or plasmid	Description	Source or reference
Strains		
B. cenocepacia J2315	ET-12 lineage, CF isolate, LMG 16656^T^	Lab collection (30)
B. cenocepacia K56-2	ET-12 lineage, CF isolate, LMG 18863	Lab collection (30)
E. coli DH5α	Propagation of replicative plasmids	Lab collection
E. coli DH5α λ*pir*	Propagation of suicide plasmids with *ori*_R6K_	Lab collection
J2315 Δ*ncS25*	J2315, *ncS25* deletion mutant	This study
J-ΔBCAL3473	J2315, BCAL3473 deletion mutant	This study
K-ΔBCAL3473	K56-2, BCAL3473 deletion mutant	This study
J-WT-VC	J2315 + pSCrhaM2	23
K-WT-VC	K56-2 + pSCrhaM2	This study
Δ*ncS25*-VC	J2315 Δ*ncS25 *+ pSCrhaM2	This study
Δ*ncS25+*pncS25	J2315 Δ*ncS25 *+ pncS25	This study
Δ*ncS25+*pncS25mut	J2315 Δ*ncS25 *+ pncS25mut	This study
J-ΔBCAL3473-VC	J2315 ΔBCAL3473 + pSCrhaM2	This study
J-ΔBCAL3473-compl	J2315 ΔBCAL3473 + pL3473	This study
K-ΔBCAL3473-VC	K56-2 ΔBCAL3473 + pSCrhaM2	This study
K-ΔBCAL3473-compl	K56-2 ΔBCAL3473 + pL3473	This study
BCAL0461 knockdown	J2315, pSC200-L0461	This study
J-WT-mCh-pos	J2315 + pSCmCh-pos	
Δ*ncS25*-mCh-pos	J2315 Δ*ncS25 *+ pSCmCh-pos	
J-WT-mCh-neg	J2315 + pSCmCh-neg	
J-WT-mChL3473	J2315 + pSCmChL3473	
Δ*ncS25*-mChL3473	J2315 Δ*ncS25 *+ pSCmChL3473	
Plasmids		
pSCrhaB2	Expression vector, *ori*_pBBR1_, *rhaR*, *rhaS*, *P_rhaB_*, *dhfr*	56
pSCrhaM2	Derivative of pSCrhaB2, SD sequence and start codons removed	38
pncS25	pSCrhaM2 with ncS25 including 167 nt upstream sequence	This study
pncS25-mut	Derivative of pncS25 with point mutations in the predicted interaction region	This study
pL3473	pSCrhaB2 with porin BCAL3473 under a rhamnose-inducible promoter	This study
pGPI-SceI-XCm	Suicide vector for allelic replacement, I-SceI restriction site, *ori*_R6K_, *dhfr*, Cm^r^	53
pDAI-SceI-SacB	I-SceI nuclease, Tet^r^, counterselectable marker SacB, *ori*_pBBR1_	53
pSC200	Suicide vector for promoter exchange, *ori*_R6K_, *rhaR*, *rhaS*, *P_rhaB_*, *dhfr*	57
pSC200-L0461	pSC200 with fragment of regulator BCAL0461	This study
pRK2013	Helper plasmid for triparental mating, *ori_colE1_*_,_ RK2 derivative, Km^r^, *mob^+^*, *tra^+^*	55
pSCmCh-pos	pSCrhaB2 with fluorescent protein mCherry	This study
pSCmCh-neg	pSCrhaM2 with mCherry	This study
pSCmChL3473	pSCrhaM2 with mCherry fused to the 5′ UTR and partial coding sequence of BCAL3473	This study

Initial screening for possible substrates of porin BCAL3473 was performed with Biolog carbon source plates PM1 and PM2A and with B. cenocepacia strain K56-2, which is from the same clonal complex as strain J2315 (30). Strain J2315 produced a uniformly low signal in all test wells of the Biolog plates and in the negative-control well and was therefore not used for this assay.

**FIG 4:**
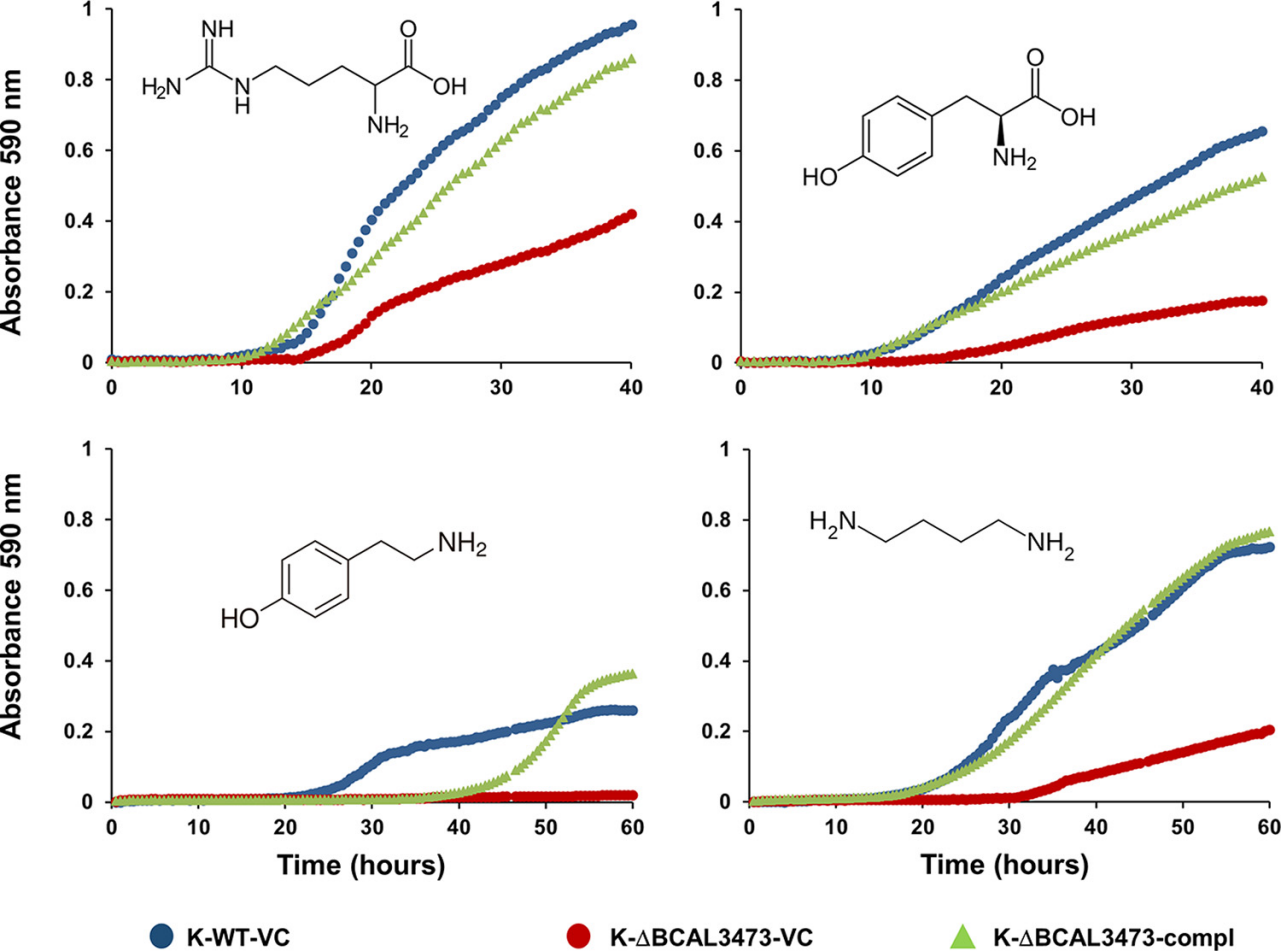
Deletion of porin BCAL3473 affects growth of B. cenocepacia K56-2 on nitrogen sources arginine, tyrosine, tyramine, and putrescine. All four compounds support growth of B. cenocepacia K56-2 when used as sole source of nitrogen. Deleting BCAL3473 attenuated or, in the case of tyramine, abolished growth. Expressing BCAL3473 in *trans* complements the phenotypes. Growth was tested in a mineral medium without ammonium and with 25 mM glucose as the carbon source. Arginine (top left), tyrosine (top right), tyramine (bottom left), and putrescine (bottom right) were added as nitrogen sources at a concentration of 2 mM. Results for one of three biological replicates are shown. WT, wild type; VC, vector control; compl, expression of BCAL3473 in *trans*.

Tyramine (the product of decarboxylation of tyrosine), arginine, and putrescine were the only substrates on these plates for which deletion mutant K-ΔBCAL3473 showed strongly reduced respiration, complemented by expressing BCAL3473 in *trans* (Table S2). To test further compounds and to confirm the Biolog results, we determined growth curves in an ammonium-free mineral medium with glucose as the carbon source and a variety of nitrogen compounds as the sole source of nitrogen, or with compounds other than glucose as the sole source of carbon. Growth of K-ΔBCAL3473 was attenuated on tyrosine, which is not included in the Biolog plates that were used, and on arginine, tyramine, and putrescine. All these phenotypes could be complemented (Fig. 4).

10.1128/msphere.00083-23.2 TABLE S2 Biolog results for wild-type B. cenocepacia K56-2, the porin BCAL3473 deletion mutant, and the complemented deletion mutant, as absorbance at 590 nm, measured after 24 h. Download Table S2, DOCX file, 0.04 MB. Copyright © 2023 Sass and Coenye. 2023 Sass and Coenye. https://creativecommons.org/licenses/by/4.0/ This content is distributed under the terms of the Creative Commons Attribution 4.0 International license.

Strain J2315 did not grow on tyrosine and tyramine as sole sources of nitrogen, possibly because of a point mutation in *hmgA* (31). Instead, it produced a brown pigment when either of these compounds was present in the medium (32). Growth with putrescine or arginine as the sole source of nitrogen was attenuated in J-ΔBCAL3473 (Fig. S2) and slightly increased with putrescine in Δ*ncS25*.

Growth on all other major amino acids, including l-ornithine and l-homoserine, and on purine and pyrimidine bases was not affected by deletion of porin BCAL3473. Utilization of the aromatic compounds 4-hydroxybenzoate, phenylacetic acid, and 4-hydroxyphenylacetic acid, as well as phenylpropionic acid and 4-hydroxyphenylpropionic acid, was unaffected by porin deletion. Utilization of the nitrogen compounds urea, ammonium, and diethanolamine was also unaffected (data not shown). The MICs of the antibiotics amikacin, tobramycin, azithromycin ciprofloxacin, chloramphenicol, tetracycline, imipenem, meropenem, and ceftazidime for J-ΔBCAL3473 did not change compared to those for the wild-type strain (Table S3). Porin protein BCAL3473 is therefore likely not involved in transporting these compounds across the OM.

10.1128/msphere.00083-23.3 TABLE S3 MICs for B. cenocepacia J2315 and the BCAL3473 deletion mutants, in Mueller-Hinton broth. Download Table S3, DOCX file, 0.02 MB. Copyright © 2023 Sass and Coenye. 2023 Sass and Coenye. https://creativecommons.org/licenses/by/4.0/ This content is distributed under the terms of the Creative Commons Attribution 4.0 International license.

## DISCUSSION

In the present study, we aimed to elucidate the biological role of NcS25, a conserved small RNA highly expressed in B. cenocepacia biofilms (22). Computational analysis of the genome context and of putative targets with complementarity to the NcS25 sequence pointed to a role in regulating porin expression. NcS25 is encoded adjacent to a porin throughout the genera in which NcS25 occurs. A conserved syntenic context points to affiliation to a certain regulon or biological response, and sRNAs and their flanking genes are often involved in the same metabolic process or stress response (14). Even if NcS25 does not regulate the porin adjacent to which it is encoded, both might still be part of the same regulatory circuit. The relatively high number of porin proteins among the targets predicted with high probability also pointed to a role of NcS25 in OM porin protein regulation, as many well-investigated sRNAs regulate multiple genes with similar function (25).

While several predicted target porins were tested for differential regulation in Δ*ncS25*, only one of these changed expression. This is not surprising, as computational target prediction based on sequence complementarity often results in many false-positive hits (25). On the other hand, since *trans*-encoded sRNAs often have multiple targets, it is likely that other genes besides BCAL3473 are targeted by NcS25, either by base-pairing or other mechanisms, and possibly under different physiological conditions.

Levels of BCAL3473 mRNA were drastically increased when *ncS25* was deleted from the genome; the large fold change increase shows that NcS25 is effective at repressing the expression of porin BCAL3473. The predicted interaction region is relatively large, longer than for other typical sRNAs which are involved in posttranscriptional regulation of porin proteins. It is also larger than that for the other predicted targets, which is the underlying reason for the large jump in CopraRNA *P* values from the first on the list to the next. RhyB, an sRNA regulating OM proteins in E. coli, needs only a 7-bp pairing region to effectively repress its targets (33). A 7-nt region complementary to the SD sequence in the target mRNA could be the seed of the interaction, as this region is located in a single-stranded part of the folded NcS25. This seed could then initiate a larger interaction, even though this would involve opening part of the secondary structure of NcS25. The free energy left after unfolding and hybridization shows that this is possible.

The conservation of the putative interaction points to a conserved mechanism of action that includes inhibiting translation initiation by occluding the SD. It is possible that the relatively long predicted interaction of NcS25 with the BCAL3473 mRNA in B. cenocepacia also induces mRNA degradation by forming an RNA-RNA duplex and thereby recruiting RNase E to the target mRNA (34), which would help to explain the large effect NcS25 has on BCAL3473 mRNA expression.

In comparison to the large effect that *ncS25* deletion has on the mRNA level, the effect seen in translational reporter gene fusions appears to be relatively small. It is likely that the abundance of NcS25 is not large enough compared to that of the target supplied in *trans* from the plasmid to allow for a bigger effect in this assay. Ideally, the sRNA is also oversupplied in assays like this, which was not possible in this case for lack of suitable vectors. Nonetheless, despite the small difference, the results from this assay confirm that BCAL3473 is regulated by NcS25.

Besides NcS25, other factors also regulate BCAL3473 expression. BCAL3473 is activated by LysR-type regulator BCAL0461, which makes it inversely regulated to the homologous porin in B. glumae, where the regulator GvmR acts as a repressor (28).

Porin BCAL3473 is upregulated under nitrogen depletion, independently of the presence of NcS25. This observation might be linked to the similarity of the BCAL3473 promoter region with the conserved σ^54^ binding site. Sigma subunits of RNA polymerase are required for selective promoter recognition and transcription initiation. The σ^54^ subunit (RpoN) forms the σ^54^ RNA polymerase holoenzyme, which binds to conserved −12 and −24 promoter elements (35). The σ^54^ holoenzyme represses expression from promoters until ATP hydrolysis by an enhancer binding protein (such as NtrC) activates transcription (36).

In B. cenocepacia H111, sigma binding sites were enriched in the promoter regions of genes induced by nitrogen limitation (29); the σ^54^ factor therefore has a role in B. cenocepacia in the response to nitrogen limitation. BCAL3473 was one of the genes upregulated by nitrogen limitation in strain H111; moreover, the porin was upregulated in a NtrC deletion mutant (27).

The BCAL3473 promoter region contains elements with the same conserved −12 and −24 sequence elements as in the consensus sequence for σ^54^-dependent binding of B. cenocepacia, but with an extended spacer sequence. Spacer length between the two conserved elements is normally conserved, a longer spacer could disrupt binding and impair repression by σ^54^ holoenzyme (37). Our results, together with the observations in B. cenocepacia strain H111, suggest that σ^54^ holoenzyme could bind, though possibly inefficiently, to the promoter region of BCAL3473.

However, expression of porin BCAL3473 does not respond specifically to nitrogen limitation. Porin BCAL3473 was constitutively expressed in LB broth and other rich media (38, 39), in line with an incomplete σ^54^-dependent repression. Likewise, NcS25 was constitutively expressed, and no evidence for differential expression was found under the conditions tested. sRNAs are often expressed only under certain stress conditions (14, 15), but this does not seem to be the case for NcS25. NcS25 could constitutively downregulate porin BCAL3473 and thus contribute to an equilibrium of porins present in the OM and to homeostasis of cell metabolism, important for fitness of the bacterium.

The outer membrane permeability of *Burkholderia* spp. for antibiotics is relatively low; in Burkholderia cepacia, it was found to be approximately 10 times lower than that of E. coli and similar to that of P. aeruginosa (40). The properties of *Burkholderia* porins should therefore promote membrane impermeability. The major porin isolated from B. cepacia, OpcP (BCAM1931 in B. cenocepacia J2315 and Opm38 in Burkholderia thailandensis and Burkholderia pseudomallei [41]), is a member of the large family of homologous porins and is relatively small (40, 42). BCAM1931 is the porin with the highest expression in B. cenocepacia J2315, as shown by RNA sequencing (38), and could therefore also be the major porin in this species. Moreover, BCAM1931 is essential in several *Burkholderia* spp. (43), which makes it the only bacterial porin protein shown to be essential for growth in several independent studies using transposon mutagenesis combined with high-throughput sequencing (43–46). The high expression and the essentiality of BCAM1931 suggest that it has an additional biological role besides its function as an OM channel, possibly in maintaining OM integrity (43).

Porin BCAL3473 mRNA is more than 250-fold less abundant than BCAM1931 (38), in part due to the repression by NcS25. It is involved in arginine and tyrosine utilization and can also transport biological amines tyramine and putrescine. Aromatic compounds similar to tyrosine and tyramine, but without amine groups, were not affected. Thus, porin BCAL3473 seems to be specific for amine-containing compounds with a variety of structures. Porins are passive diffusion channels and often not specific for a single compound (4). On the other hand, amino acids such as arginine can be transported by more than one porin, as observed in P. aeruginosa (4, 47). This could also be the case in B. cenocepacia, since the porin deletion mutants were still growing on arginine and tyrosine, albeit more slowly. In B. cenocepacia K-56-2 porin BCAL3474 appears to be essential for growth on tyramine, and in B. cenocepacia J2315 for growth on putrescine. The accelerated growth of the Δ*ncS25* mutant on putrescine shows that increased expression of BCAL3473 can accelerate transport of this compound. These two compounds could therefore be specifically transported by porin BCAL3473.

### Conclusion.

The present study represents, to the best of our knowledge, the first characterization of the substrate specificity of an OM porin protein in B. cenocepacia. Porin BCAL3473 is involved in transport of arginine, tyrosine, tyramine, and putrescine across the OM, which makes it an important part of nitrogen metabolism in B. cenocepacia. Porin BCAL3473 is constitutively expressed and simultaneously strongly repressed by NcS25, possibly to dampen unwanted translation that would otherwise lead to imbalances in porins present in the OM. Based on the conservation of the interaction regions between sRNA and porin mRNA, this regulation appears to be conserved throughout several genera of *Burkholderiaceae*, suggesting that the downregulation of BCAL3473 by NcS25 is important for fitness in these bacteria. The regulation of porins is probably as complex in B. cenocepacia as in other bacteria, and this study is just the first step toward elucidating the specificity and regulation of the many porins present in B. cenocepacia.

## MATERIALS AND METHODS

### Strains and media.

Strains and plasmids used in this study are listed in Table 2. Strains were routinely cultured in LB broth or agar supplemented with antibiotics when necessary (50 μg/mL trimethoprim, 50 kanamycin, or 20 μg/mL tetracycline for Escherichia coli strains; 800 μg/mL trimethoprim or 250 μg/mL tetracycline for B. cenocepacia strains). For experiments, a phosphate-buffered mineral medium was used, either with or without ammonium (2 g/L NH_4_Cl, 4.25 g/L K_2_HPO_4_·3H_2_O [ChemLab], 1 g/L NaH_2_PO_4_·H_2_O, 0.1 g/L nitrilotriacetic acid, 0.003 g/L MnSO_4_·H_2_O, 0.003 g/L ZnSO_4_·7H_2_O, 0.001 g/L CoSO_4_·7H_2_O, 0.2 g/L MgSO_4_·7H_2_O, and 0.012 g/L FeSO_4_·7H_2_O [Sigma-Aldrich]). Organic components were 5 g/L glycerol (Scharlab), 5 g/L yeast extract (Lab M), and 2 g/L Bacto peptone (BD Biosciences) when a medium with high concentrations of carbohydrates and amino acids was required (referred to as rich mineral medium). To grow strains under nitrogen depletion, mineral medium without ammonium was supplemented with 25 mM glucose as the carbon source. Media were supplemented with 600 μg/mL (strain J2315) or 200 μg/mL (strain K56-2) trimethoprim as the selective antibiotic when appropriate. Gene expression from plasmids was induced by adding rhamnose (Sigma) to a final concentration of 0.2% (wt/vol). All incubations were performed at 37°C.

### Computational methods.

Homologous sRNA and 5′ UTR sequences were searched for using BLASTn (48), with the following adapted parameters: word size, 7; match/mismatch score, 3/−2; gap existence cost, 2; gap extension cost, 2. Homologous porin genes were screened for using BLASTp and the *Burkholderia* genome database (12). Genes were defined as homologous to BCAL3473 at >95% query coverage and >60% amino acid identity and defined as belonging to the same porin family at >80% query coverage and >30% amino acid identity.

Computational target prediction was performed with CopraRNA (25), using default parameters. This algorithm takes accessibility of interaction sites and conservation of putative targets into account. Sequences from 200 nt upstream to 100 nt downstream of the first nucleotide of annotated genes were considered for target prediction. Only genes which were conserved in at least 50% of the input strains were reported as output. Input strains were B. cenocepacia J2315, Burkholderia ambifaria AMMD, Burkholderia dolosa AU0158, Burkholderia gladioli BSR3, Burkholderia glumae BGR1, Burkholderia lata sp. 383, Burkholderia multivorans ATCC 17616, Burkholderia pyrrocinia DSM 10685, Burkholderia thailandensis E264, Burkholderia vietnamiensis G4, Paraburkholderia caribensis DSM 13236, Paraburkholderia fungorum BAA-463, Paraburkholderia graminis PHS1, Paraburkholderia hospita DSM 17164, Paraburkholderia phenoliruptrix BR3459a, Paraburkholderia phymatum STM815, Paraburkholderia phytofirmans PsJN, Paraburkholderia terrae DSM 17804, Paraburkholderia terricola mHS1, Paraburkholderia xenovorans LB400, Pandoraea apista TF80G25, Pandoraea faecigallinarum DSM 23572, Pandoraea fibrosis 7641, Pandoraea norimbergensis DSM 11628, Pandoraea oxalativorans DSM 23570, Pandoraea pnomenusa TF-18, Pandoraea pulmonicola DSM 16583, Pandoraea sputorum DSM 21091, Pandoraea vervacti NS15, and Pandoraea thiooxydans DSM 25325.

sRNA secondary structures were predicted with RNAfold (49). Alignments of sRNAs and 5′ UTRs of BCAL3473 and their homologs were computed with the LocARNA program (50), using the strains listed above. The results from LocARNA were then used as input for the RILogo software (51) to create sequence logos depicting conservation. Interactions between sRNAs and mRNAs were predicted with the IntaRNA tool (52).

### Construction of mutants.

*ncS25* was deleted from the B. cenocepacia J2315 genome by a method based on allelic recombination (53). Inserts flanking the sequence region targeted for deletion were amplified using PrimeSTAR GXL high-fidelity DNA polymerase (TaKaRa Bio) and cloned into the suicide vector pGPI-SceI-XCm. After successful recombination, pGPI-SceI-XCm was removed from the genome by introducing a double-strand break using the endonuclease Sce-I from the vector pDAI-SceI-SacB, followed by homologous recombination. Deletion mutants were screened for by PCR using primers annealing adjacent to the deleted sequence. Correct insert sequences and DNA junctions were confirmed by Sanger sequencing. All transformations of B. cenocepacia strains were performed by triparental mating (54, 55).

Porin BCAL3473 was deleted from B. cenocepacia J2315 and K56-2 using the same method, resulting in mutants J-ΔBCAL3473 and K-ΔBCAL3473.

J-ΔBCAL3473 and K-ΔBCAL3473 were complemented by cloning the entire gene into vector pSCrhaB2 (56), where its expression is under the control of a rhamnose-inducible promoter, resulting in vector pL3473.

For complementing Δ*ncS25*, *ncS25*, including 167 nt directly upstream and presumably containing the native promoter, was cloned into a modified version of pSCrhaB2 without the start codon and SD sequence, pSCrhaM2 (38), resulting in plasmid pncS25. This construct was then used as the template for further modifications by reverse PCR of the entire plasmid, resulting in pncS25mut, with point mutations in the predicted interaction region.

A knockdown mutant of regulator BCAL0461 was constructed by cloning a 376-nt fragment of the 5′ end of BCAL0461 into the suicide vector pSC200 (57). The resulting plasmid, pSC200-L0461, was transformed into strain J2315, and ex-transformants were screened for correct insertion by PCR using primers annealing upstream of the BCAL0461 fragment in the vector and downstream of the fragment in the genome.

To construct a translational reporter gene fusion, the 5′ UTR and the start of the coding sequence of BCAL3473 were fused to the fluorescent protein mCherry and cloned into the one available plasmid usable for cloning and in *trans* expression in B. cenocepacia. The fused protein would probably be transported into the periplasm, because the target sequence contains a transport tag. mCherry, unlike green fluorescent protein (GFP), is stable in the periplasm.

The entire gene for fluorescent protein mCherry was amplified from pmCherry (Clontech) and cloned into pSCrhaB2, resulting in plasmid pSCmCh-pos, which was used as a positive control for fluorescence protein production. mCherry without a start codon was amplified and cloned into pSCrhaM2, resulting in plasmid pSCmCh-neg, which was used for translational fusions and for negative controls. The entire 5′ UTR and the first 84 nt of the BCAL3473 gene were cloned into plasmid pSCmCh-neg, resulting in plasmid pSCmChL3473. All constructs are under the control of a rhamnose-inducible promoter. The three plasmids were transformed into wild-type J2315 and its Δ*ncS25* mutant. Plasmid pSCmCh-neg was used as a negative control for fluorescence production without fused gene fragments.

Amplification of all plasmid inserts was performed with PrimeSTAR GXL high-fidelity DNA polymerase (TaKaRa Bio). The sequences of all plasmid inserts were verified by Sanger sequencing. All primer sequences are listed in Table S4.

10.1128/msphere.00083-23.4 TABLE S4 Primers used in the present study. Download Table S4, DOCX file, 0.02 MB. Copyright © 2023 Sass and Coenye. 2023 Sass and Coenye. https://creativecommons.org/licenses/by/4.0/ This content is distributed under the terms of the Creative Commons Attribution 4.0 International license.

### Biolog screen and growth curves in microtiter plates.

Biolog PM1 and PM2A plates were used as carbon sources according to the manufacturer’s instruction with modifications. Cells were grown over night in LB broth in the presence of trimethoprim, washed, and resuspended in physiological saline (0.9% [wt/vol] NaCl). Cells were resuspended in IF-0 inoculating fluid to a final optical density (OD) of 0.1, corresponding to 10^8^ CFU/mL. Biolog redox dye mix A (1×) was added as a redox indicator and rhamnose (0.2% [wt/vol]) was added as an inducer to the IF-0 inoculating fluid in all experiments, and 100 μL per well was added to the Biolog plates. Absorption was read at 580 nm in a microplate reader after 24 h of incubation at 37°C (Victor Nivo, Perkin Elmer).

Growth on compounds as sole sources of nitrogen was tested in mineral medium lacking ammonium and containing 25 mM glucose as the carbon source. Nitrogen-containing compounds were added at a 2 mM final concentration. Growth on compounds as sole sources of carbon was tested in mineral medium containing ammonium. The test compound was added at a 5, 10, or 20 mM final concentration depending on the molecular weight and solubility of the compound, aiming at approximately 60 mM carbon equivalents.

Growth curves were determined in round-bottom 96-well microtiter plates, filled with 200 μL medium per well. Bacterial strains were grown overnight to an OD of 1.0 in the mineral medium without ammonium on 25 mM glucose and 0.5% Bacto peptone, to minimize nitrogen compound carryover, and then diluted to an OD of 0.001 (10^6^ CFU/mL).

Absorption was read at 600 nm every 30 min in a microplate reader set to 37°C (Victor Nivo, Perkin Elmer).

The MICs of antibiotics were determined in Mueller-Hinton broth in 96-well plates in duplicate, according to the EUCAST broth microdilution protocol. Wells with visible turbidity were scored as positive.

### Translational reporter gene fusion assay.

Mutant strains were grown overnight in mineral medium containing 2 g/L of each glucose and glycerol, supplemented with trimethoprim. The cultures were harvested by centrifugation and washed with the same mineral medium without trimethoprim. Cultures were normalized to an OD of 1.0, and 100 μL was added to the wells of a microtiter plate. One hundred microliters of LB broth with rhamnose to a final concentration of 0.1% was added to each well. The plate was incubated at 37°C, and fluorescence (excitation wavelength, 555 nm, and emission wavelength, 635 nm) was determined every 20 min using a microtiter plate reader (Envision multilabel reader; PerkinElmer).

### qPCR.

For evaluating gene expression in the Δ*ncS25* strain and the BCAL0461 knockdown mutant, cells were grown in rich mineral medium. Glass flasks (250 mL) with 25 mL medium were inoculated with a liquid overnight culture to approximately 4 × 10^7^ CFU/mL and incubated in a shaking incubator at 100 rpm until an OD of 0.5 was reached.

For evaluating the response to nitrogen starvation, strains were grown overnight in LB and then centrifuged, washed, and resuspended in mineral medium without ammonium. The washed cells were set to an OD of 1.0 and supplemented with either 25 mM glucose (nitrogen-depleted condition) or 25 mM glucose, 35 mM ammonium chloride, and 0.5% Bacto peptone (nitrogen-replete condition). Twenty-five milliliters of cells was further incubated in flasks on a shaker and harvested after 3 h.

Cells were snap cooled, pelleted by centrifugation at 4°C and stored at −80°C for a maximum of 1 week. RNA extraction, cDNA generation, and qPCR were performed as described previously (23). *C_q_* values were normalized to that of the control gene *rpoD* (BCAM0918), which had shown stable expression in previous studies (22–24).

One-way analysis of variance (ANOVA) with a Tukey *post hoc* test using SPSS v. 25 was performed to determine statistical significance. If variances were not equal, a nonparametric Kruskal-Wallis test was applied.

Primer sequences are listed in Table S4.
