# Supplementary figures and images for: The Small RNA NcS25 Regulates Biological Amine-Transporting Outer Membrane Porin BCAL3473 in Burkholderia cenocepacia
Source: mSphere. 2023 Mar 27;8(2):e00083-23. doi: 10.1128/msphere.00083-23 (PMC10117139; doi:10.1128/msphere.00083-23)

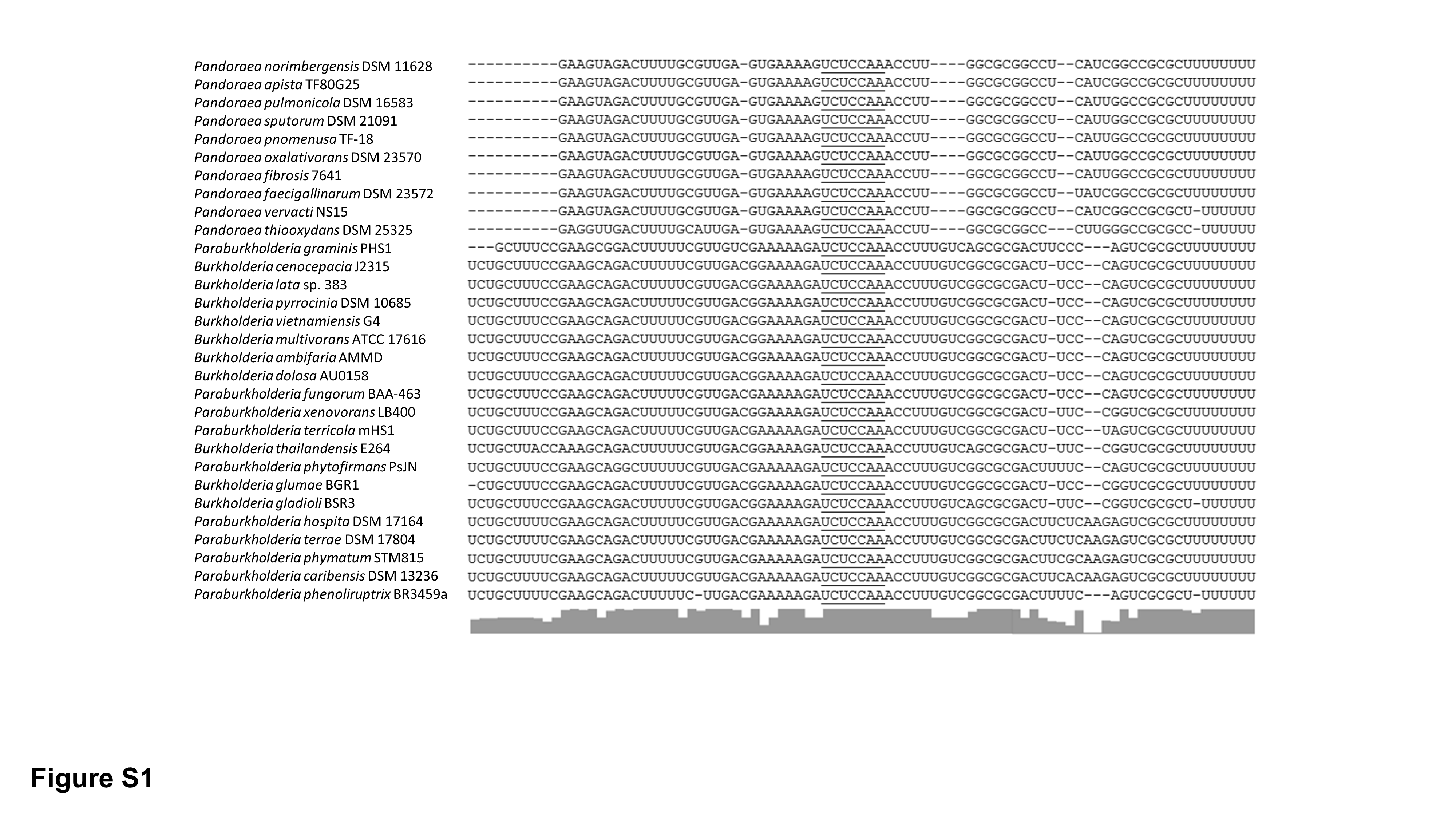

Supplement: FIG S1 [file msphere.00083-23-s0005.tif]

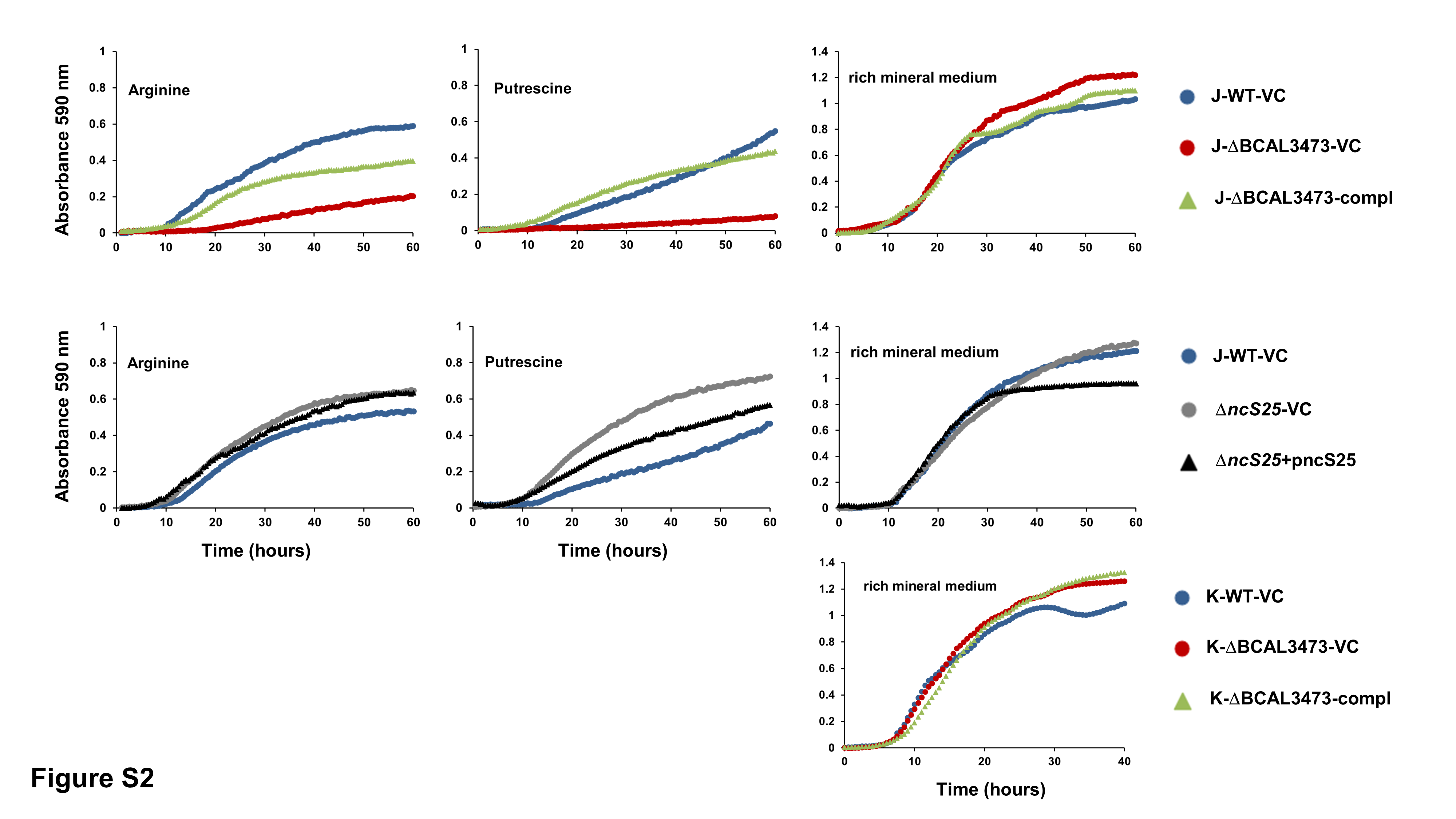

Supplement: FIG S2 [file msphere.00083-23-s0006.tif]

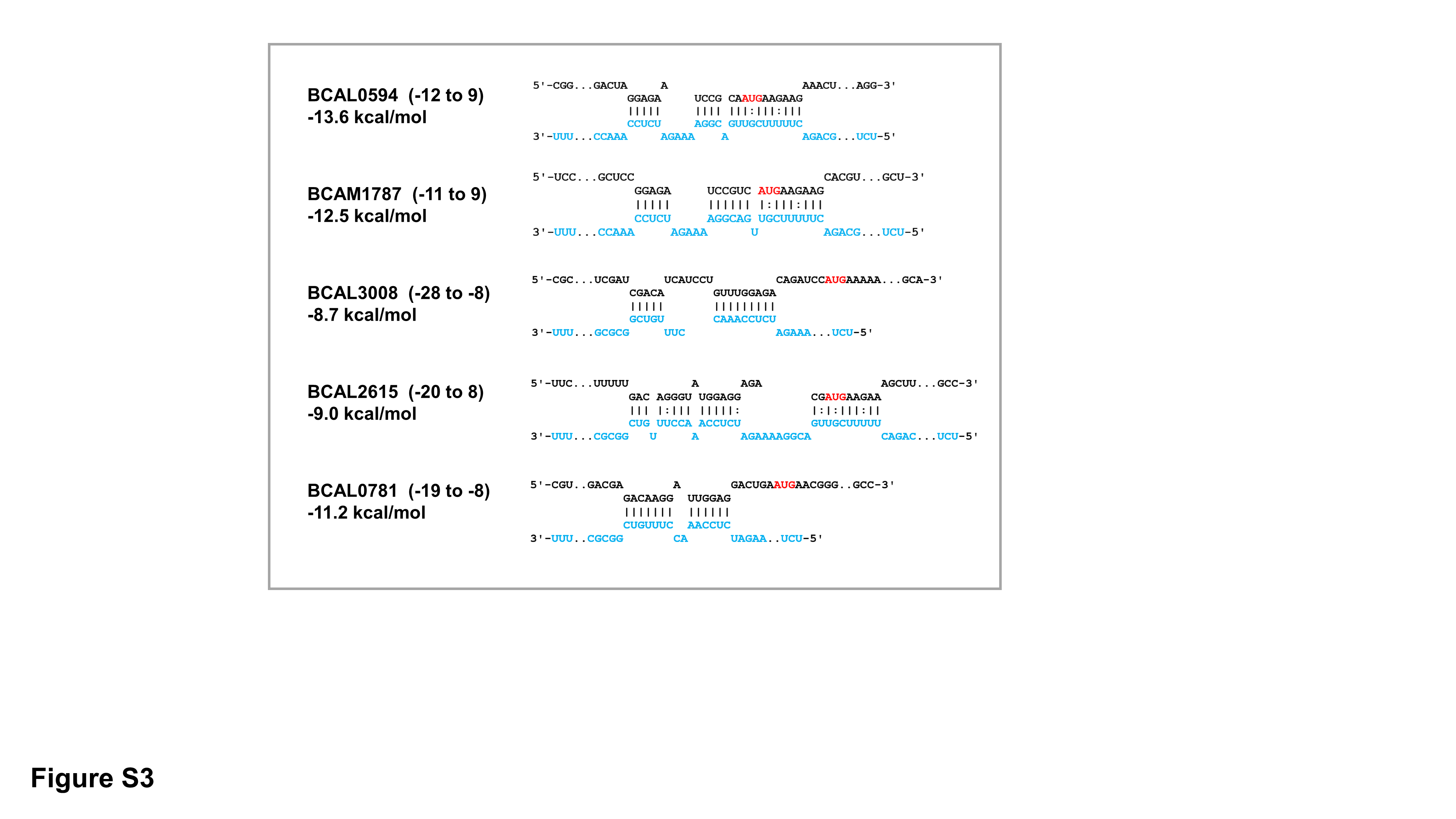

Supplement: FIG S3 [file msphere.00083-23-s0007.tif]

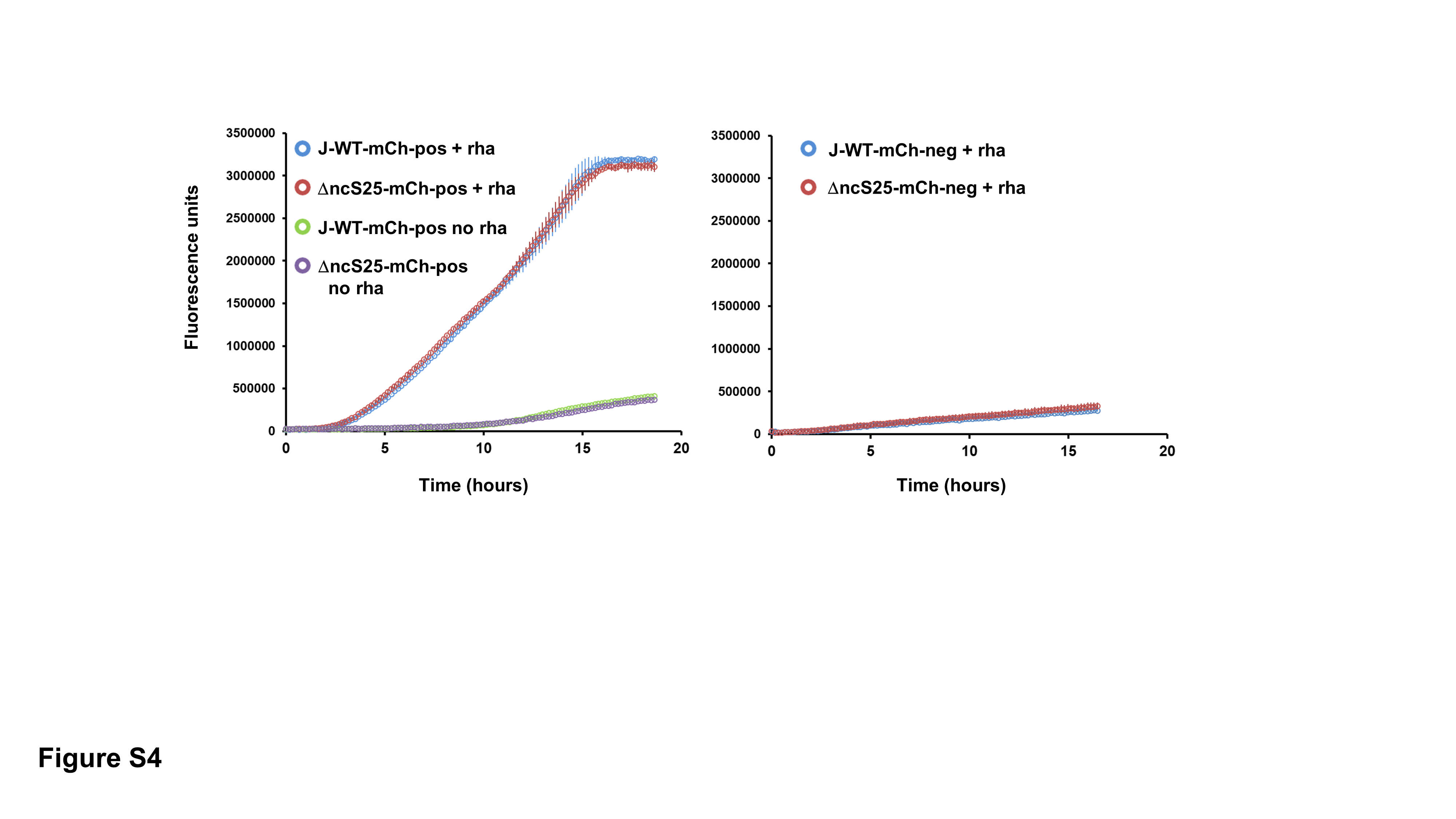

Supplement: FIG S4 [file msphere.00083-23-s0008.tif]
