# Supplementary material for: The Small RNA NcS25 Regulates Biological Amine-Transporting Outer Membrane Porin BCAL3473 in Burkholderia cenocepacia
Source: mSphere. 2023 Mar 27;8(2):e00083-23. doi: 10.1128/msphere.00083-23 (PMC10117139; doi:10.1128/msphere.00083-23)
Supplement: TABLE S2 [file msphere.00083-23-s0002.docx]

**Table S2: Biolog results for *B. cenocepacia* K56-2 wild type, porin BCAL3473 deletion mutant and complemented deletion mutant, as absorbance at 590 nm, measured after 24 hours.**

|  |  |  |  | ***B. cenocepacia* K56-2** | **K-ΔBCAL3473** | **K-ΔBCAL3473-compl** |
| --- | --- | --- | --- | --- | --- | --- |
|  |  |  |  | **Wild type** |  |  |
|  |  |  |  |  |  |  |
| **Plate** | **Location** | **Chemical** | **KEGG ID** |  |  |  |
| PM01 | A01 | Negative Control |  | 0.206 | 0.22 | 0.218 |
| PM01 | A02 | L-Arabinose | C00259 | 0.907 | 1.028 | 1.098 |
| PM01 | A03 | N-Acetyl-D-Glucosamine | C00140 | 0.197 | 0.23 | 0.248 |
| PM01 | A04 | D-Saccharic acid | C00818 | 1.024 | 1.054 | 1.097 |
| PM01 | A05 | Succinic acid | C00042 | 0.966 | 0.928 | 1.113 |
| PM01 | A06 | D-Galactose | C00124 | 1.076 | 0.997 | 1.021 |
| PM01 | A07 | L-Aspartic acid | C00049 | 1.314 | 0.907 | 1.114 |
| PM01 | A08 | L-Proline | C00148 | 1.113 | 1.018 | 0.888 |
| PM01 | A09 | D-Alanine | C00133 | 0.536 | 0.618 | 0.613 |
| PM01 | A10 | D-Trehalose | C01083 | 0.822 | 0.361 | 0.356 |
| PM01 | A11 | D-Mannose | C00159 | 0.557 | 0.691 | 0.812 |
| PM01 | A12 | Dulcitol | C01697 | 0.922 | 0.701 | 0.91 |
| PM01 | B01 | D-Serine | C00740 | 0.197 | 0.159 | 0.197 |
| PM01 | B02 | D-Sorbitol | C00794 | 1.182 | 1.114 | 1.055 |
| PM01 | B03 | Glycerol | C00116 | 0.531 | 0.584 | 0.524 |
| PM01 | B04 | L-Fucose | C01019 | 0.835 | 0.882 | 1.1 |
| PM01 | B05 | D-Glucuronic acid | C00191 | 0.534 | 0.27 | 0.267 |
| PM01 | B06 | D-Gluconic acid | C00257 | 1.455 | 1.136 | 1.022 |
| PM01 | B07 | DL-a-Glycerol Phosphate | C00093 | 0.577 | 0.675 | 0.724 |
| PM01 | B08 | D-Xylose | C00181 | 0.57 | 0.719 | 0.524 |
| PM01 | B09 | L-Lactic acid | C00186 | 0.802 | 0.954 | 1.011 |
| PM01 | B10 | Formic acid | C00058 | 0.395 | 0.44 | 0.718 |
| PM01 | B11 | D-Mannitol | C00392 | 1.082 | 1.093 | 1.145 |
| PM01 | B12 | L-Glutamic acid | C00025 | 1.107 | 1.082 | 1.016 |
| PM01 | C01 | D-Glucose-6-Phosphate | C00092 | 0.832 | 1.074 | 1.036 |
| PM01 | C02 | D-Galactonic acid-g-Lactone | C03383 | 0.97 | 1.028 | 1.211 |
| PM01 | C03 | DL-Malic acid | C00497 | 1.154 | 1.086 | 1.236 |
| PM01 | C04 | D-Ribose | C00121 | 0.331 | 0.401 | 0.549 |
| PM01 | C05 | Tween 20 | C11624 | 0.782 | 1.133 | 1.025 |
| PM01 | C06 | L-Rhamnose | C00507 | 0.151 | 0.17 | 0.16 |
| PM01 | C07 | D-Fructose | C00095 | 1.116 | 1.085 | 1.029 |
| PM01 | C08 | Acetic acid | C00033 | 0.687 | 0.666 | 1.044 |
| PM01 | C09 | a-D-Glucose | C00267 | 1.328 | 1.163 | 1.069 |
| PM01 | C10 | Maltose | C00208 | 0.189 | 0.247 | 0.237 |
| PM01 | C11 | D-Melibiose | C05402 | 0.186 | 0.232 | 0.251 |
| PM01 | C12 | Thymidine | C00214 | 0.285 | 0.317 | 0.351 |
| PM01 | D01 | L-Asparagine | C00152 | 1.053 | 1.016 | 0.841 |
| PM01 | D02 | D-Aspartic acid | C00402 | 0.215 | 0.237 | 0.401 |
| PM01 | D03 | D-Glucosaminic acid | C03752 | 0.298 | 0.493 | 0.749 |
| PM01 | D04 | 1,2-Propanediol | C00583 | 0.157 | 0.178 | 0.178 |
| PM01 | D05 | Tween 40 |  | 0.666 | 0.736 | 1.281 |
| PM01 | D06 | a-Ketoglutaric acid | C00026 | 0.347 | 0.356 | 0.55 |
| PM01 | D07 | a-Ketobutyric acid | C00109 | 0.483 | 0.44 | 0.193 |
| PM01 | D08 | a-Methyl-D-Galactoside |  | 0.244 | 0.212 | 0.233 |
| PM01 | D09 | a-D-Lactose | C00243 | 0.22 | 0.246 | 0.254 |
| PM01 | D10 | Lactulose | C07064 | 0.166 | 0.213 | 0.221 |
| PM01 | D11 | Sucrose | C00089 | 1.225 | 1.05 | 0.72 |
| PM01 | D12 | Uridine | C00299 | 0.162 | 0.187 | 0.187 |
| PM01 | E01 | L-Glutamine | C00064 | 1.271 | 0.941 | 1.044 |
| PM01 | E02 | m-Tartaric acid | C00552 | 0.244 | 0.265 | 0.402 |
| PM01 | E03 | D-Glucose-1-Phosphate | C00103 | 0.61 | 0.675 | 0.716 |
| PM01 | E04 | D-Fructose-6-Phosphate | C00085 | 0.998 | 1.15 | 1.094 |
| PM01 | E05 | Tween 80 | C11625 | 0.57 | 0.661 | 0.611 |
| PM01 | E06 | a-Hydroxyglutaric acid-g-Lactone |  | 0.485 | 0.603 | 0.843 |
| PM01 | E07 | a-Hydroxybutyric acid | C05984 | 0.431 | 0.442 | 0.523 |
| PM01 | E08 | b-Methyl-D-Glucoside |  | 0.197 | 0.236 | 0.312 |
| PM01 | E09 | Adonitol | C00474 | 0.873 | 0.978 | 0.931 |
| PM01 | E10 | Maltotriose | C01835 | 0.24 | 0.319 | 0.361 |
| PM01 | E11 | 2`-Deoxyadenosine | C00559 | 0.239 | 0.277 | 0.333 |
| PM01 | E12 | Adenosine | C00212 | 0.181 | 0.207 | 0.369 |
| PM01 | F01 | Gly-Asp | C02871 | 0.243 | 0.278 | 0.375 |
| PM01 | F02 | Citric acid | C00158 | 1.279 | 1.074 | 1.203 |
| PM01 | F03 | m-Inositol | C00137 | 0.773 | 0.989 | 0.983 |
| PM01 | F04 | D-Threonine | C00820 | 0.192 | 0.184 | 0.208 |
| PM01 | F05 | Fumaric acid | C00122 | 1.195 | 1.097 | 1.195 |
| PM01 | F06 | Bromosuccinic acid |  | 0.504 | 0.625 | 1.026 |
| PM01 | F07 | Propionic acid | C00163 | 0.741 | 0.61 | 0.547 |
| PM01 | F08 | Mucic acid | C01807 | 1.061 | 1.062 | 0.999 |
| PM01 | F09 | Glycolic acid | C00160 | 0.498 | 0.486 | 0.317 |
| PM01 | F10 | Glyoxylic acid | C00048 | 0.212 | 0.229 | 0.206 |
| PM01 | F11 | D-Cellobiose | C00185 | 0.221 | 0.273 | 0.174 |
| PM01 | F12 | Inosine | C00294 | 0.232 | 0.262 | 0.182 |
| PM01 | G01 | Gly-Glu |  | 0.242 | 0.297 | 0.184 |
| PM01 | G02 | Tricarballylic acid |  | 1.122 | 0.938 | 1.064 |
| PM01 | G03 | L-Serine | C00065 | 0.996 | 1.108 | 1.132 |
| PM01 | G04 | L-Threonine | C00188 | 0.391 | 0.714 | 0.903 |
| PM01 | G05 | L-Alanine | C00041 | 0.982 | 0.791 | 0.925 |
| PM01 | G06 | Ala-Gly |  | 0.341 | 0.695 | 1.151 |
| PM01 | G07 | Acetoacetic acid | C00164 | 0.263 | 0.258 | 0.2 |
| PM01 | G08 | N-Acetyl-D-Mannosamine | C00645 | 0.178 | 0.209 | 0.173 |
| PM01 | G09 | Mono-Methylsuccinate |  | 0.238 | 0.29 | 0.356 |
| PM01 | G10 | Methylpyruvate |  | 0.561 | 0.946 | 1.165 |
| PM01 | G11 | D-Malic acid | C00497 | 0.574 | 0.455 | 0.978 |
| PM01 | G12 | L-Malic acid | C00149 | 0.856 | 0.844 | 1.242 |
| PM01 | H01 | Gly-Pro |  | 0.807 | 0.665 | 0.93 |
| PM01 | H02 | p-Hydroxyphenyl Acetic acid | C00642 | 0.823 | 0.621 | 0.829 |
| PM01 | H03 | m-Hydroxyphenyl Acetic acid | C05593 | 0.686 | 0.524 | 0.456 |
| PM01 | H04 | Tyramine | C00483 | 0.411 | 0.225 | 0.873 |
| PM01 | H05 | D-Psicose | C06468 | 0.227 | 0.305 | 0.176 |
| PM01 | H06 | L-Lyxose | C01508 | 0.134 | 0.14 | 0.159 |
| PM01 | H07 | Glucuronamide | C13295 | 0.168 | 0.211 | 0.163 |
| PM01 | H08 | Pyruvic acid | C00022 | 1.038 | 1.1 | 1.235 |
| PM01 | H09 | L-Galactonic acid-g-Lactone | C01115 | 0.263 | 0.504 | 0.523 |
| PM01 | H10 | D-Galacturonic acid | C00333 | 1.025 | 0.755 | 0.819 |
| PM01 | H11 | Phenylethylamine | C05332 | 0.577 | 0.475 | 0.214 |
| PM01 | H12 | 2-Aminoethanol | C00189 | 0.414 | 0.365 | 0.226 |
| PM02A | A01 | Negative Control |  | 0.193 | 0.238 | 0.217 |
| PM02A | A02 | Chondroitin Sulfate C | C00635 | 0.179 | 0.249 | 0.264 |
| PM02A | A03 | a-Cyclodextrin |  | 0.176 | 0.231 | 0.249 |
| PM02A | A04 | b-Cyclodextrin |  | 0.178 | 0.22 | 0.221 |
| PM02A | A05 | g-Cyclodextrin |  | 0.165 | 0.237 | 0.248 |
| PM02A | A06 | Dextrin | C00721 | 0.198 | 0.291 | 0.29 |
| PM02A | A07 | Gelatin | C01498 | 0.181 | 0.225 | 0.255 |
| PM02A | A08 | Glycogen | C00182 | 0.191 | 0.242 | 0.366 |
| PM02A | A09 | Inulin | C03323 | 0.219 | 0.314 | 0.299 |
| PM02A | A10 | Laminarin | C00771 | 0.28 | 0.345 | 0.292 |
| PM02A | A11 | Mannan | C00464 | 0.152 | 0.225 | 0.231 |
| PM02A | A12 | Pectin | C00714 | 0.733 | 0.429 | 0.371 |
| PM02A | B01 | N-Acetyl-D-Galactosamine | C01132 | 0.272 | 0.274 | 0.308 |
| PM02A | B02 | N-Acetyl-Neuraminic acid | C00270 | 0.132 | 0.189 | 0.197 |
| PM02A | B03 | b-D-Allose | C01487 | 0.139 | 0.188 | 0.194 |
| PM02A | B04 | Amygdalin | C08325 | 0.161 | 0.226 | 0.22 |
| PM02A | B05 | D-Arabinose | C00216 | 0.909 | 0.701 | 0.287 |
| PM02A | B06 | D-Arabitol | C01904 | 1.097 | 0.82 | 1.022 |
| PM02A | B07 | L-Arabitol | C00532 | 1.042 | 0.924 | 1.012 |
| PM02A | B08 | Arbutin | C06186 | 0.749 | 1.07 | 1.083 |
| PM02A | B09 | 2-Deoxy-D-Ribose | C01801 | 0.794 | 0.711 | 0.904 |
| PM02A | B10 | i-Erythritol | C00503 | 0.158 | 0.217 | 0.294 |
| PM02A | B11 | D-Fucose | C01018 | 0.193 | 0.286 | 0.259 |
| PM02A | B12 | 3-O-b-D-Galactopyranosyl-D-Arabinose |  | 0.513 | 0.441 | 0.285 |
| PM02A | C01 | Gentiobiose | C08240 | 0.161 | 0.227 | 0.292 |
| PM02A | C02 | L-Glucose |  | 0.146 | 0.183 | 0.201 |
| PM02A | C03 | D-Lactitol |  | 0.167 | 0.239 | 0.222 |
| PM02A | C04 | D-Melezitose | C08243 | 0.181 | 0.229 | 0.233 |
| PM02A | C05 | Maltitol | G00275 | 0.163 | 0.216 | 0.216 |
| PM02A | C06 | a-Methyl-D-Glucoside |  | 0.166 | 0.226 | 0.26 |
| PM02A | C07 | b-Methyl-D-Galactoside | C03619 | 0.493 | 0.336 | 0.274 |
| PM02A | C08 | 3-Methylglucose |  | 0.213 | 0.223 | 0.316 |
| PM02A | C09 | b-Methyl-D-Glucuronic acid | C08350 | 0.17 | 0.211 | 0.249 |
| PM02A | C10 | a-Methyl-D-Mannoside |  | 0.173 | 0.24 | 0.297 |
| PM02A | C11 | b-Methyl-D-Xyloside |  | 0.156 | 0.222 | 0.248 |
| PM02A | C12 | Palatinose | C01742 | 0.16 | 0.18 | 0.138 |
| PM02A | D01 | D-Raffinose | C00492 | 0.161 | 0.223 | 0.271 |
| PM02A | D02 | Salicin | C01451 | 0.486 | 0.682 | 0.524 |
| PM02A | D03 | Sedoheptulosan |  | 0.166 | 0.223 | 0.254 |
| PM02A | D04 | L-Sorbose | C00247 | 0.169 | 0.214 | 0.221 |
| PM02A | D05 | Stachyose | C01613 | 0.161 | 0.215 | 0.296 |
| PM02A | D06 | D-Tagatose | C00795 | 0.346 | 0.284 | 0.298 |
| PM02A | D07 | Turanose | G03588 | 0.193 | 0.253 | 0.287 |
| PM02A | D08 | Xylitol | C00379 | 1.012 | 0.895 | 0.831 |
| PM02A | D09 | N-Acetyl-D-Glucosaminitol |  | 0.17 | 0.168 | 0.192 |
| PM02A | D10 | g-Amino-N-Butyric acid | C00334 | 0.861 | 0.577 | 0.612 |
| PM02A | D11 | d-Amino Valeric acid |  | 0.179 | 0.142 | 0.115 |
| PM02A | D12 | Butyric acid | C00246 | 1.271 | 0.727 | 0.918 |
| PM02A | E01 | Capric acid | C01571 | 0.483 | 0.333 | 0.292 |
| PM02A | E02 | Caproic acid | C01585 | 1.289 | 0.997 | 0.979 |
| PM02A | E03 | Citraconic acid | C02226 | 1.494 | 0.765 | 0.65 |
| PM02A | E04 | Citramalic acid | C00815 | 0.836 | 0.685 | 0.646 |
| PM02A | E05 | D-Glucosamine | C00329 | 0.634 | 0.524 | 0.514 |
| PM02A | E06 | 2-Hydroxybenzoic acid | C00805 | 0.107 | 0.11 | 0.141 |
| PM02A | E07 | 4-Hydroxybenzoic acid | C00156 | 1.116 | 0.589 | 0.623 |
| PM02A | E08 | b-Hydroxybutyric acid | C01089 | 1.367 | 1.132 | 1.263 |
| PM02A | E09 | g-Hydroxybutyric acid | C00989 | 0.223 | 0.293 | 0.271 |
| PM02A | E10 | a-Keto-Valeric acid | C00567 | 0.206 | 0.326 | 0.164 |
| PM02A | E11 | Itaconic acid | C00490 | 0.187 | 0.186 | 0.149 |
| PM02A | E12 | 5-Keto-D-Gluconic acid | C01062 | 0.355 | 0.41 | 0.221 |
| PM02A | F01 | D-Lactic acid Methyl Ester |  | 0.192 | 0.275 | 0.329 |
| PM02A | F02 | Malonic acid | C00383 | 0.793 | 0.9 | 1.133 |
| PM02A | F03 | Melibionic acid |  | 0.119 | 0.203 | 0.245 |
| PM02A | F04 | Oxalic acid | C00209 | 0.183 | 0.216 | 0.24 |
| PM02A | F05 | Oxalomalic acid | C01990 | 0.24 | 0.272 | 0.263 |
| PM02A | F06 | Quinic acid | C00296 | 1.301 | 1.089 | 1.165 |
| PM02A | F07 | D-Ribono-1,4-Lactone |  | 0.164 | 0.214 | 0.248 |
| PM02A | F08 | Sebacic acid | C08277 | 1.135 | 0.859 | 1.072 |
| PM02A | F09 | Sorbic acid |  | 0.727 | 0.546 | 0.43 |
| PM02A | F10 | Succinamic acid |  | 0.235 | 0.319 | 0.385 |
| PM02A | F11 | D-Tartaric acid | C02107 | 0.191 | 0.245 | 0.265 |
| PM02A | F12 | L-Tartaric acid | C00898 | 0.753 | 0.632 | 0.817 |
| PM02A | G01 | Acetamide | C06244 | 0.278 | 0.335 | 0.316 |
| PM02A | G02 | L-Alaninamide |  | 0.235 | 0.285 | 0.318 |
| PM02A | G03 | N-Acetyl-L-Glutamic acid | C00624 | 0.179 | 0.217 | 0.183 |
| PM02A | G04 | L-Arginine | C00062 | 1.374 | 0.852 | 1.284 |
| PM02A | G05 | Glycine | C00037 | 0.378 | 0.347 | 0.232 |
| PM02A | G06 | L-Histidine | C00135 | 1.105 | 1.076 | 1.257 |
| PM02A | G07 | L-Homoserine | C00263 | 0.146 | 0.1 | 0.15 |
| PM02A | G08 | Hydroxy-L-Proline | C01015 | 1.66 | 1.055 | 1.058 |
| PM02A | G09 | L-Isoleucine | C00407 | 0.262 | 0.359 | 0.386 |
| PM02A | G10 | L-Leucine | C00123 | 0.335 | 0.392 | 0.365 |
| PM02A | G11 | L-Lysine | C00047 | 0.819 | 0.886 | 1.026 |
| PM02A | G12 | L-Methionine | C00073 | 0.133 | 0.175 | 0.2 |
| PM02A | H01 | L-Ornithine | C00077 | 0.253 | 0.64 | 0.269 |
| PM02A | H02 | L-Phenylalanine | C00079 | 1.304 | 0.955 | 1.03 |
| PM02A | H03 | L-Pyroglutamic acid | C02238 | 1.388 | 0.915 | 1.089 |
| PM02A | H04 | L-Valine | C00183 | 0.224 | 0.33 | 0.248 |
| PM02A | H05 | D,L-Carnitine | C00487 | 0.161 | 0.217 | 0.153 |
| PM02A | H06 | sec-Butylamine |  | 0.204 | 0.171 | 0.154 |
| PM02A | H07 | D,L-Octopamine | C04227 | 0.946 | 0.725 | 0.951 |
| PM02A | H08 | Putrescine | C00134 | 0.811 | 0.298 | 0.505 |
| PM02A | H09 | Dihydroxyacetone | C00184 | 0.164 | 0.177 | 0.189 |
| PM02A | H10 | 2,3-Butanediol | C03044 | 0.325 | 0.474 | 0.246 |
| PM02A | H11 | 2,3-Butanedione | C00741 | 0.154 | 0.177 | 0.191 |
| PM02A | H12 | 3-Hydroxy-2-butanone | C00466 | 0.18 | 0.237 | 0.259 |
