# Supplementary material for: The Small RNA NcS25 Regulates Biological Amine-Transporting Outer Membrane Porin BCAL3473 in Burkholderia cenocepacia
Source: mSphere. 2023 Mar 27;8(2):e00083-23. doi: 10.1128/msphere.00083-23 (PMC10117139; doi:10.1128/msphere.00083-23)
Supplement: TABLE S3 [file msphere.00083-23-s0003.docx]

**Table S3: MIC values for *B. cenocepacia* J2315 and the BCAL3473 deletion mutants, in MH broth.**

|  | J-WT-VC | J‑ΔBCAL3473-VC | J‑ΔBCAL3473-comp |
| --- | --- | --- | --- |
| Amikacin | 512 | 512 | 512 |
| Tobramycin | 256 | 256 | 256 |
| Erythromycin | 256 | 256 | 256 |
| Azithromycin | 64 | 64 | 64 |
| Chloramphenicol | 32 | 64 | 32 |
| Tetracycline | 64 | 128 | 64 |
| Ciprofloxacin | 4 | 4 | 4 |
| Meropenem | 32 | 32 | 32 |
| Imipenem | 256 | 256 | 256 |
| Ceftazidime | 64 | 64 | 64 |
