# Supplementary material for: The Small RNA NcS25 Regulates Biological Amine-Transporting Outer Membrane Porin BCAL3473 in Burkholderia cenocepacia
Source: mSphere. 2023 Mar 27;8(2):e00083-23. doi: 10.1128/msphere.00083-23 (PMC10117139; doi:10.1128/msphere.00083-23)
Supplement: TABLE S4 [file msphere.00083-23-s0004.docx]

**Table S4: Primers used in the present study**

| **Gene deletion** |  |
| --- | --- |
| preS25-BglII | AATAGATCTAATCTTCACCGCCATTGTCC |
| preS25-NheI | AAAGCTAGCCTTTGTCGGCGCGACTTC |
| postS25-NheI | TTTGCTAGCTCCATTTCATTTCGCTGCAC |
| postS25-ApaI | TTTGGGCCCCACGGTTGTTCGAGAACTGG |
| join-S25F | TGCGATCCTGAATCAACACC |
| join-S25R | AAGCCATGTGCAGTGTACGG |
| UL3473-F-SmaI | AATCCCGGGTGCTCGTCATGACTTTCGAC |
| UL3473-R-BglII | AATAGATCTTTAAACCATGCTGTACAGGTGCTTG |
| DL3473-F-BglII | AATAGATCTACAACGCGAACACGTACCAG |
| DL3473-R-NheI | AAAGCTAGCATCCGAACGAAGTCGAGGAC |
| join-L3473F | CCTGCTCTTCAACAACAACG |
| join-L3473R | TGCGCTTCGACAGGAAGTAG |
| **Complementation** |  |
| L3473ovF-NdeI | AAAATTATATACATATGAAAAAGTCGCTTCTCGCGCTC |
| L3473ovR-XbaI | AAATCTAGAGACGCACGTTTGCATGTCGAC |
| S25Ov -SpeI-F1 | AATACTAGTGCAGCGAAATGAAATGGAAC |
| S25Ov-HindIII-R1 | AATAAGCTTCCGCTCGTACACGCAGTAG |
| S25-mutF | GCAATAGTCTGCTTCGGAAAGCAG |
| S25-mutR | GTTGACGTAATAGATCTGGAAACCTTTGTCG |
| **BCAL0461 knock-down mutant** |  |
| L0461_C_Nde | AAAAAAATACATATGGATCATTTGCAGTCGATGCG |
| L0461_C_Xba | AATTCTAGATGAGATCCGGCACCACCTTC |
| rha-F | CACGTTCATCTTTCCCTGGT |
| L0461int | AGCCTTCCTCGACGAGATC |
| **Translational fusions** |  |
| mCherry-NdeI-F | TTTTTTTTTTCATATGGTGAGCAAGGGCGAGGAGG |
| mCherry-BamHI-R | AATGGATCCTTATTACTTGTACAGCTCGTCCATGCC |
| mCherry-XbaI-F | ATATTCTAGAGTGAGCAAGGGCGAGGAGG |
| L3473-KpnI-F | AAAGGTACCACGTGCGACTTCTTGCGAAGTTGATGG |
| L3473-XbaI-R | AAATCTAGAGCCGTAAAGCGTCACGCTGCTTTG |
| **qPCR (forward / reverse)** |  |
| ncS25 | CCGAAGCAGACTTTTTCGTTG / GCCGACAAAGGTTTGGAGA |
| ncS25mut | TTTCCGAAGCAGACTATTGC / CCGACAAAGGTTTCCAGATC |
| BCAM0918 (RpoD), control gene | GAGATGAGCACCGATCACAC / CCTTCGAGGAACGACTTCAG |
| BCAL3473 | ACAACGCGAACACGTACCAG / TGAAGTTGATTTCGCCGTTG |
| BCAL3008 | ACCGACGTGTATGTGCAAGG / CGATCTGCTTGTTGCTCGAC |
| BCAL2615 | AAGGTGCGTTCAACACGATG / CCAACAGCAGCGTACCGTAG |
| BCAM1787 | CAACACGGACAACTCGTTCC / TGTACTGGCGGTTGTTCGAG |
| BCAL0781 | GAAGGACATCGATCCCAAGC / CGAAGAACGCGAGGTAGTCC |
| BCAL0594 | ATCACGCTGCTGCAGAACTC / TGAGCATCTGGTTCGACTGC |
| BCAL0461 | CGTGTTCGTCAAGGTTGCAG / GGCACGCTCCAGATAGACCT |
| BCAL3098 | AGCTGAACGGGCTGCTCTAC / CCGTGCTCATCAGGTACTCG |
| BCAL0729 | CGTTCAAGCTCGATGAGACG / ATCTTCGGCAGGAAATCGAC |

Restriction sites are underlined; mismatches introduced into ncS25 by primers are indicated in red
